# Improved Intranasal Retentivity and Transnasal Absorption Enhancement by PEGylated Poly-l-ornithine

**DOI:** 10.3390/ph11010009

**Published:** 2018-01-25

**Authors:** Yusuke Kamiya, Tsutomu Yamaki, Shigehiro Omori, Masaki Uchida, Kazuo Ohtake, Mitsutoshi Kimura, Hiroshi Yamazaki, Hideshi Natsume

**Affiliations:** 1 Faculty of Pharmaceutical Sciences, Josai University, 1-1 Keyakidai, Sakado, Saitama 350-0295, Japan; kamiya@ac.shoyaku.ac.jp (Y.K.); yamaki@josai.ac.jp (T.Y.); gyd1503@josai.ac.jp (S.O.); muchida@josai.ac.jp (M.U.); kazuo@josai.ac.jp (K.O.); mkimura@josai.ac.jp (M.K.); 2 Laboratory of Drug Metabolism and Pharmacokinetics, Showa Pharmaceutical University, 3-3165 Higashi-tamagawa Gakuen, Machida, Tokyo 194-8543, Japan; hyamazak@ac.shoyaku.ac.jp

**Keywords:** poly-l-ornithine (PLO), polyethylene glycol (PEG), absorption enhancer, intranasal administration, retentivity

## Abstract

We reported that the introduction of polyethylene glycol (PEG) to poly-l-ornithine (PLO), which is an homopolymeric basic amino acid having absorption-enhancement ability, prolonged retention time in an in vitro inclined plate test, probably due to an increase in viscosity caused by PEGylation. The aim of the present study is to investigate whether the introduction of PEG chains to PLO improves intranasal retention and transnasal absorption in vivo. We performed intranasal administration experiments using PLO and PEG-PLO with a model drug, fluorescein isothiocyanate dextran (FD-4), in rats under closed and open systems. In the open system, transition of plasma FD-4 concentration after co-administration with unmodified PLO was low, and the area under the plasma concentration-time curve (AUC) decreased to about 60% of that in the closed system. In contrast, the AUC after co-administration with PEG-PLO in the open system was about 90% of that in the closed system, and the transition of plasma FD-4 concentration and FD-4 absorption profile were similar to those of the closed system. These findings indicate that introducing PEG chains to homopolymeric basic amino acids (HPBAAs) is a very useful method for developing a functional absorption enhancer that can exhibit an efficient in vivo absorption-enhancing effect.

## 1. Introduction

Transnasal drug delivery has attracted attention as an alternative administration route for the administration of drugs that have low bioavailability by oral administration since the nasal mucosa has a large surface area, which is advantageous in terms of the absorption of high-molecular-weight (MW) drugs [1,2]. It was reported that homopolymeric basic amino acids (HPBAAs) such as poly-l-arginine (PLA) and poly-l-ornithine (PLO) enhanced the transnasal absorption of hydrophilic macromolecules, including peptide and protein drugs, without causing mucosal damage [3,4,5]. The absorption-enhancement mechanism of HPBAAs was considered to be due to their ability to open the paracellular pathway by changing the localization of tight junction (TJ) proteins from cellular contacts to the subcellular space [6,7]. Furthermore, the absorption-enhancing effects of HPBAAs depend on the MW and application time [8]. Hence, HPBAAs have the potential to provide an efficient transnasal drug-delivery system that can meet individual demands of unabsorbable drugs including hydrophilic macromolecules.

The nasal cavity has two clearance mechanisms, namely, mucociliary clearance by the cilia and physical clearance via its structure [9,10]. These mechanisms are important from the viewpoint of biophylaxis because they act to remove extraneous substances attached to the mucosa. However, since HPBAAs do not have mucoadhesive/retentive properties, in an experimental system in which the administered solution can flow out from the nasal cavity to the pharynx side, a drug solution containing HPBAAs administered to the nasal cavity is removed by these clearance mechanisms from the absorption area. Therefore, effective transnasal absorption of drugs corresponding to the dose of HPBAAs has not been obtained. Thus, it is important not only to increase the absorption rate, but also to improve intranasal retentivity, in order to improve the bioavailability of transnasal administration.

We previously reported that in vitro retentivity of PLO increased by introducing polyethylene glycol (PEG), compared with that of unmodified-PLO [11]. This increase in retentivity was presumed to be due to an increase in viscosity caused by PEGylation, as reported in studies on the PEGylation of protein [12], suggesting that the retentivity of PEGylated-PLO (PEG-PLO) might also be improved in vivo. However, we reported that PEG-PLO is difficult to apply to intranasal administration experiments because its viscosity is too high [11]. It has been reported that the increase in viscosity achieved by PEGylation depends on the MW and the modification number of PEG to be modified [12]. In the present study, we synthesized PEG-PLO having a different MW ratio of PEG and PLO from that previously reported [11] and evaluated the in vitro retentivity- and the hydrophilic macromolecules permeation-enhancing ability of PEG-PLO using Caco-2 cell sheet, which is known to have a good correlation between permeability across the sheet and the nasal absorbability in rats [13], and used this for evaluation of the permeation-enhancing effects and mechanisms of HPBAAs [7,14]. Moreover, the improvement in retentivity in the nasal cavity by PEGylation was estimated pharmacokinetically using an “in vivo intranasal retentivity evaluation system” [15]. Furthermore, we also investigated the effects of PEGylation on the localization of TJ proteins, which play an important role in the mechanisms of absorption enhancement by HPBAAs.

## 2. Results

### 2.1. Synthesis of PEGylated-PLO (PEG-PLO)

**Figure 1:**
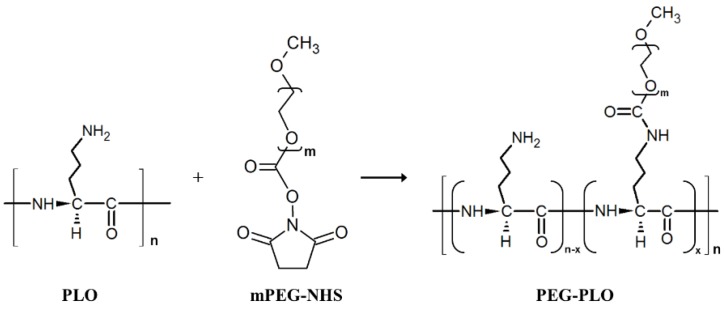
Synthesis scheme of polyethylene glycol modified poly-l-ornithine (PEG-PLO) using *N*-hydroxysuccinimide (mPEG-NHS). PLO and mPEG-NHS were mixed in a 1:10 molar ratio. The mixture was stirred overnight at room temperature (r.t.).

**Figure 2:**
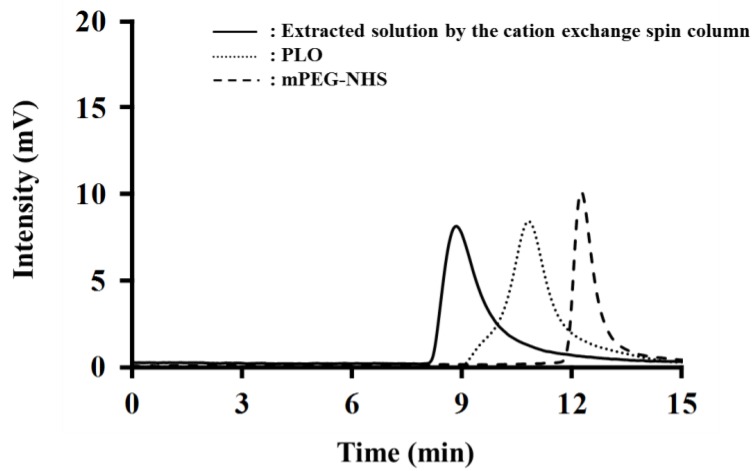
Typical chromatogram of size-exclusion high-performance liquid chromatography (HPLC). After reaction and extraction (solid line), a new peak derived from PEG-PLO was observed earlier than the PLO (dotted line) and mPEG-NHS (dashed line) peaks, and the PLO and mPEG-NHS peaks were not detected.

PEG-PLO synthesis was carried out by an amine/*N*-hydroxysuccinimide (NHS) ester reaction between the side chain of PLO and mPEG-NHS, as indicated in Figure 1. The product was confirmed by size exclusion high-performance liquid chromatography (HPLC). Figure 2 shows typical chromatograms of PLO, mPEG-NHS and solution extracted by a spin column containing PEG-PLO. Before the reaction, PLO (dotted line) and mPEG-NHS (dashed line) peaks were observed at 10.84 min and 12.28 min, respectively. After the reaction followed by spin column extraction, a new peak (8.87 min, solid line, Figure 2) was detected earlier than the peaks derived from PLO and mPEG-NHS, and the peaks were not observed after the spin column extraction, indicating that PEG-PLO having a larger MW than the reactants had been produced and there was no unreacted PLO or mPEG-NHS in the reaction solution. Therefore, only PEG-PLO could be extracted by these methods.

### 2.2. Determination of the PEGylation Ratio of PEG-PLO

**Figure 3:**
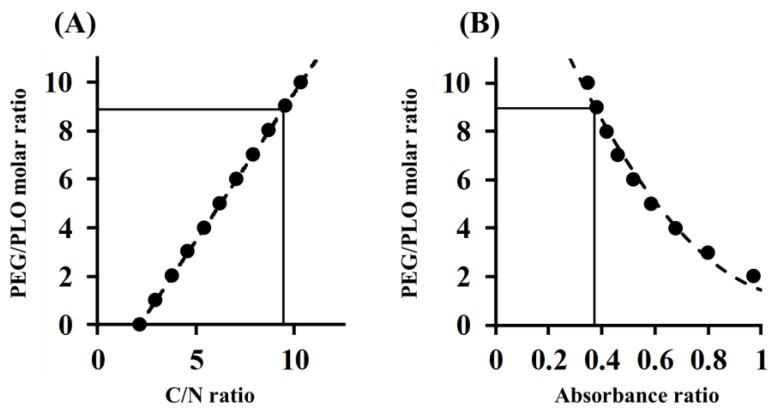
Determination of PEG/PLO molar ratio of PEG-PLO by elemental analysis (**A**); and TNBS (2,4,6-Trinitrobenzenesulfonic acid) assay (**B**). ●: theoretical value; ----: regression line.

In order to determine the PEGylation ratio of PEG-PLO, elemental analysis and TNBS (2,4,6-Trinitrobenzenesulfonic acid) assay were performed. The carbon-to-nitrogen (C/N) ratio of PEG-PLO was measured using an elemental analysis method, and correcting values were 9.37 and 9.45, respectively. The PEGylation ratio determined by fitting to a regression line obtained from the theoretical value was 8.90 (Figure 3A and Table 1 and Table 2). The absorbance ratio of PEG-PLO and PLO determined by TNBS assay was 0.374, and the PEGylation ratio calculated by fitting to the regression line obtained from the theoretical value was 8.96 (Figure 3B and Table 2). The values obtained by both methods were similar, suggesting that the PEGylation ratios obtained were appropriate and that PEG-PLO bears an average of 8–9 PEG chains per PLO molecule (Table 2). When an approximate MW of PEG-PLO was calculated from the PEGylation ratio, and the number of primary amine groups in a side chain per 1 kDa (NH_2_ contents) was compared with that before PEGylation, the NH_2_ content was found to decrease by PEGylation (Table 2).

### 2.3. Impact of PEGylation of PLO on In Vitro Retentivity

**Figure 4:**
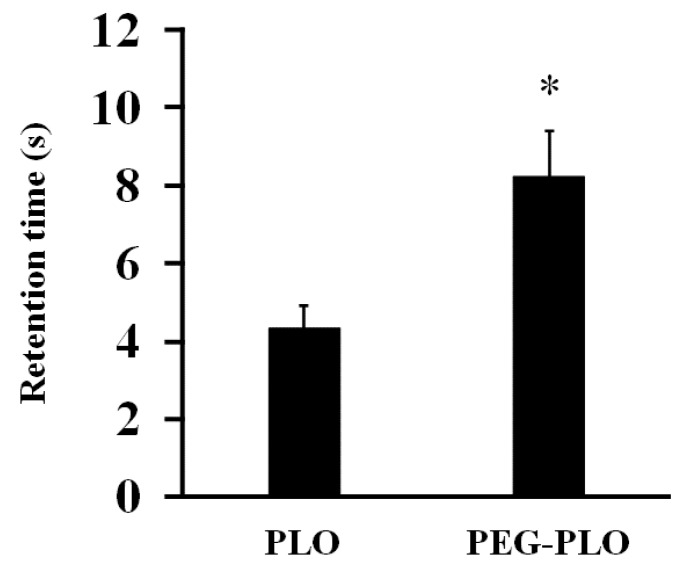
Retention time of PEG-PLO solutions in the in vitro inclined plate test. Retention time was defined as the time required for the solution to flow 3 cm. All measurements were performed at r.t. on the same day. Each data column represents the mean ± S.E. (*n* = 6), * *p* < 0.05 compared with the PLO.

The in vitro inclined plate test was carried out to evaluate the retentivity of PEG-PLO (Figure 4). A 0.1% (*w*/*v*) PLO solution took 4.33 s to flow down the stainless steel plate (SSP). In contrast, a 0.1% (*w/v*) PEG-PLO solution took 8.25 s. This prolongation of retention time by PEGylation was consistent with a previous report [11]. This in vitro result suggested the possibility that in vivo intranasal retentivity also increased by PEGylation. 

### 2.4. Effects of PEG-PLO on Fluorescein Isothiocyanate Dextran (FD-4) Permeation Across Caco-2 Cells

**Figure 5:**
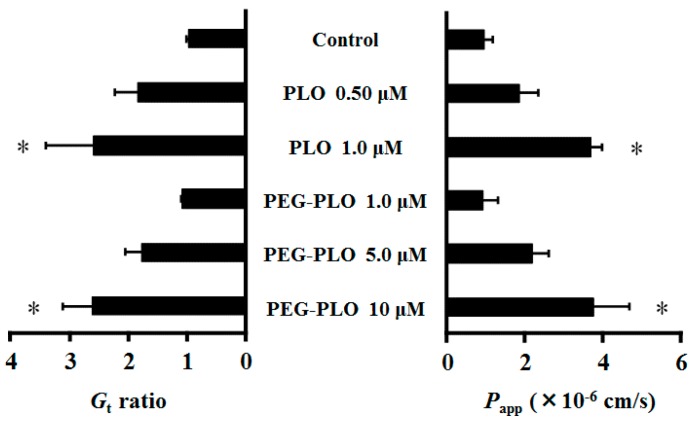
Effects of PLO and PEG-PLO on membrane conductance (*G*_t_) and permeability of fluorescein isothiocyanate dextran (FD-4) (*P*_app_) in Caco-2 cells. Relationship between *G*_t_ and *P*_app_ after application of PLO and PEG-PLO. Control group was treated with vehicle (HBSS). Each data column represents the mean ± S.E. (*n* = 3–4). * *p* < 0.05 compared with the control group.

Each *G*_t_ (membrane conductance) was expressed in the ratio of 120 to 0 min values because the permeation-enhancing effects of HPBAAs were considered to have reached a steady state [7,11]. *G*_t_ and the apparent permeability coefficient (*P*_app_) of fluorescein isothiocyanate dextran (FD-4, MW 3.4 kDa) in Caco-2 cells increased dose-dependently by application of PLO and PEG-PLO. Interestingly, the *G*_t_ and *P*_app_ of FD-4 after application of 1.0 μM PLO and 10 μM PEG-PLO were almost the same (Figure 5). The titer of the permeation-enhancing effect of PEG-PLO was 1/10 as compared with unmodified-PLO.

### 2.5. Cell Viability after Application of PEG-PLO

**Figure 6:**
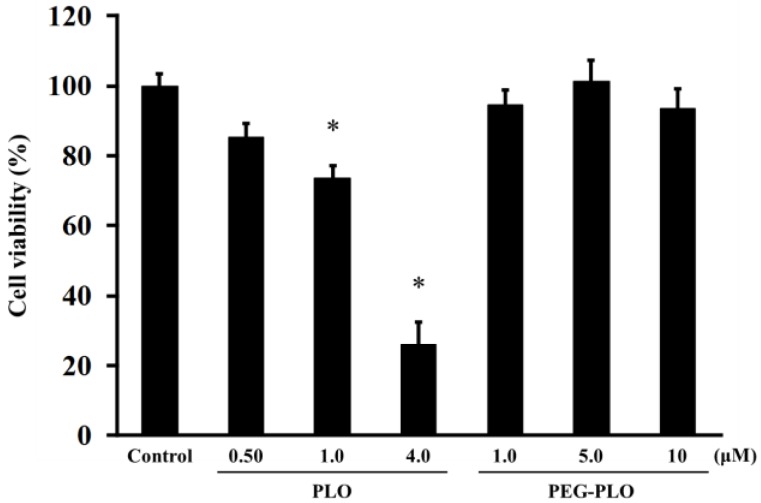
Effects of PLO and PEG-PLO on cell viability in Caco-2 cells. Control group was treated with vehicle (Hank’s balanced salt solution (HBSS)). Each data column represents the mean ± S.E. (*n* = 3–4). * *p* < 0.05 compared with the control group.

Cytotoxicities of PLO and PEG-PLO were determined by MTT (3-(4,5-dimethyl-2-thiazolyl)-2,5-diphenyl-2*H*-tetrazolium bromide) assay. The cell viabilities after application are shown in Figure 6. PLO was decreased the cell viability at 1.0 μM, a concentration that enhanced permeation. Furthermore, the viability decreased significantly at the higher concentration (4.0 μM). On the other hand, PEG-PLO showed no cytotoxicity even at the highest concentration in this study. These results suggested that the conjugation of PEG was important for reduction of the cytotoxicity of PLO.

### 2.6. Tight Junction (TJ) Protein Localization after Application of PLO and PEG-PLO

**Figure 7:**
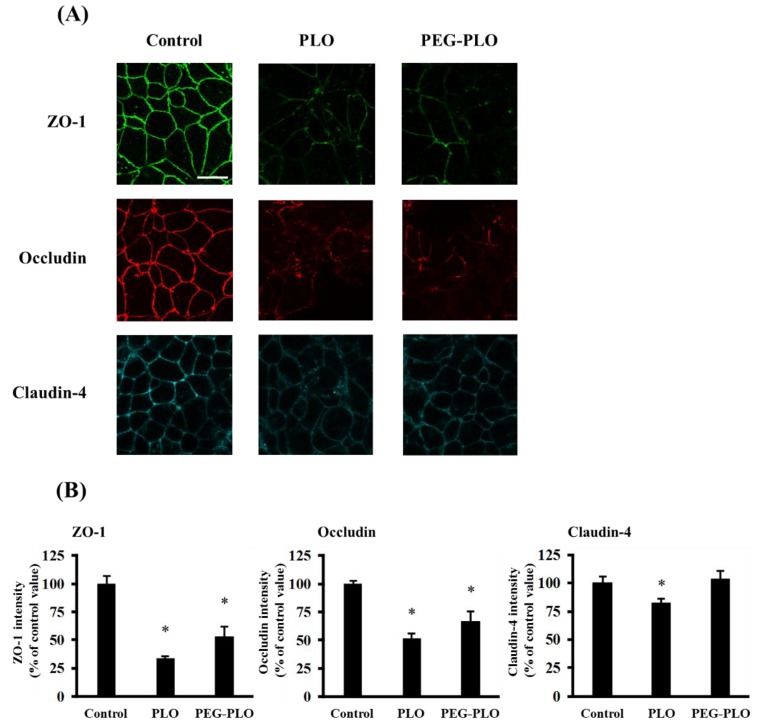
Effects of PLO and PEG-PLO on the distribution of tight junction (TJ) proteins in Caco-2 cells. The distribution of TJ proteins was visualized by immunofluorescent staining after exposure to PLO (1.0 μM) and PEG-PLO (10 μM), scale bar is 20 μm (**A**); the intensity of each protein at the paracellular space was calculated (**B**). Each data column represents the mean ± S.E. (*n* = 3), * *p* < 0.05 compared with the control group.

The distribution of TJ proteins, zonnula occludens-1 (ZO-1), occludin and claudin-4, was visualized by immunofluorescent staining after application of PLO (1.0 μM) and PEG-PLO (10 μM). Fluorescence images and intensity at the paracellular space of each protein are shown in Figure 7A,B, respectively. The intensities of TJ proteins decreased by application of PLO (the percentages of intensity of ZO-1, occludin and claudin-4 to the intensity in the control group were 33.5%, 52.0% and 82.1%, respectively). Application of PEG-PLO decreased the intensities of ZO-1 and occludin (53.0% and 67.0% to control, respectively), but the intensity of claudin-4 was maintained at a similar level in the control group.

### 2.7. Impact of PEGylation of PLO on Intranasal Retentivity and Absorption-Enhancing Effect

**Figure 8:**
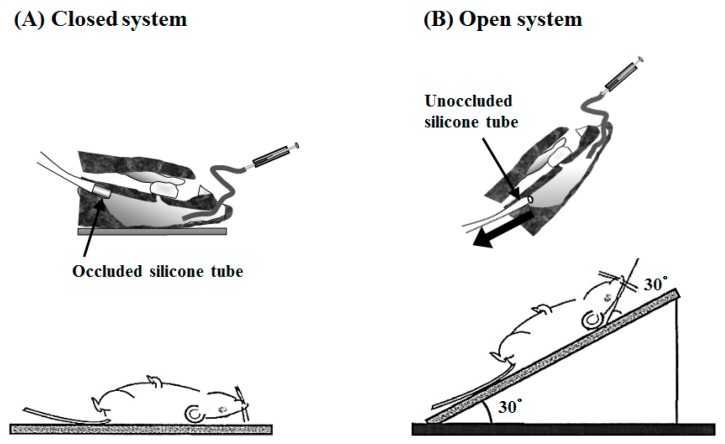
In vivo intranasal (*i.n.*) retentivity evaluation system: closed (**A**), and open (**B**), systems.

**Figure 9:**
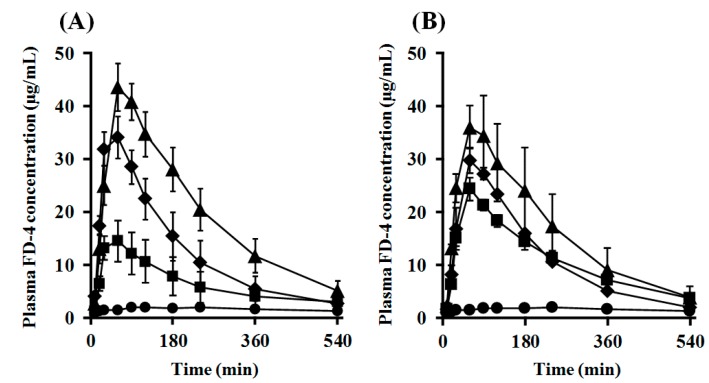
Plasma FD-4 concentration after *i.n.* administration in rats under the closed system. (**A**) PLO (●: Control, ■: 0.05%, ♦: 0.25% and ▲: 0.50%); (**B**) PEG-PLO (●: Control, ■: 0.50%, ♦: 1.00% and ▲: 2.00%). Each data point represents the mean ± S.E. (*n* = 3–7).

The intranasal (*i.n.*) administration experiments were performed using PLO and PEG-PLO in an “in vivo intranasal retentivity evaluation system”, i.e., closed and open systems (Figure 8A,B, respectively) [15], under various conditions. Transitions of plasma concentration and pharmacokinetic parameters of FD-4 after *i.n.* administration with PLO and PEG-PLO at various concentrations in the closed system, in which the dosage formulation could not leak to the esophagus side (Figure 8A), are shown in Figure 9 and Table 3, respectively. In the FD-4 alone administration group, plasma FD-4 concentration did not increase (black circle in Figure 9A,B). In contrast, in the co-administration groups with PLO and PEG-PLO, transitions of plasma FD-4 concentration increased (Figure 9A,B) and The maximum plasma FD-4 concentration (*C*_max_), AUC from 0 to 540 min (*AUC*_0–540_) and *F*_0–540_ showed dose-dependent higher values of the enhancers (Table 3). At the same concentration (0.50%), *F*_0–540_ was 71.6% in PLO, in comparison with 40.4% in PEG-PLO. PLO exhibited a significant increase in *AUC*_0–540_ at 0.25% (approximately 55.7 μM calculated from Table 2); however, PEG-PLO showed almost the same degree of absorption-enhancing effect at 1.00% (approximately 87.0 μM calculated from Table 2). The titer of absorption-enhancing effect was not decreased as much as seen in vitro. 

**Figure 10:**
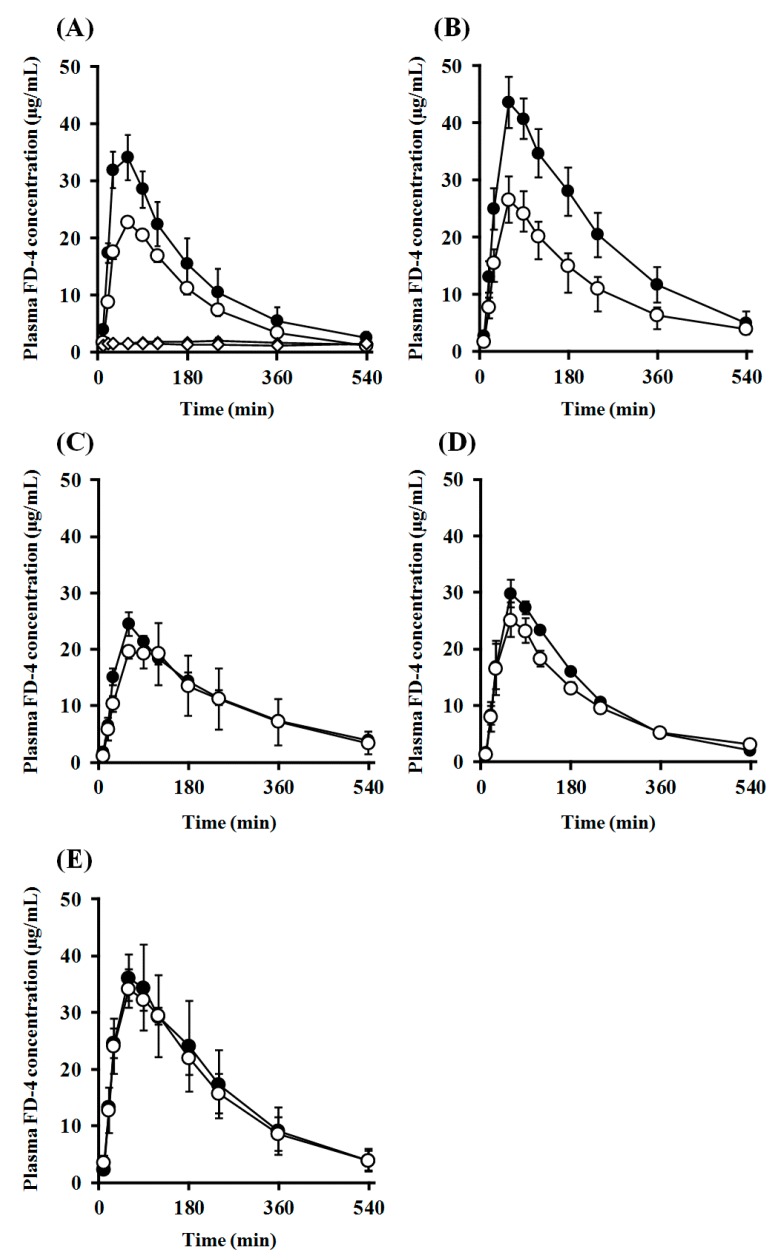
Comparison of plasma FD-4 concentration after *i.n.* administration in rats under closed and open systems; ●: Closed system, ○: Open system; (**A**): PLO (0.25%) (♦ and ◊ indicate the control group in the closed and open systems, respectively); (**B**): PLO (0.50%); (**C**): PEG-PLO (0.50%); (**D**): PEG-PLO (1.00%); (**E**): PEG-PLO (2.00%). Each data point represents the mean ± S.E. (*n* = 3–7).

Subsequently, *i.n.* administration experiments were performed in the open system, in which the dosage formulation could leak to the esophagus side (Figure 8B), and the absorption-enhancing effects in the closed and open systems were compared. Comparison of the transition of plasma concentration and pharmacokinetic parameters of FD-4 after *i.n.* administration with PLO and PEG-PLO at various concentrations between the open and closed systems are shown in Figure 10 and Table 4, respectively. In all conditions, including the FD-4 alone administration group, transitions of plasma FD-4 concentration in the open system decreased compared with those of the closed system (Figure 10), and *C*_max_, *AUC*_0–540_ and *F*_0–540_ values were also low (Table 4). The *C*_max_ values of PLO in the open system significantly decreased compared with those of the closed system at both concentrations of 0.25% and 0.50% (Table 4). Moreover, *AUC*_0–540_ of PLO at both concentrations also decreased markedly, and the retentivity improvement rate (*F*_o/c_) in 0.25% and 0.50% PLO were 66.7% and 57.5%, respectively. In contrast, transitions of plasma FD-4 concentration after *i.n.* administration with PEG-PLO in the closed and open systems were almost the same, and *F*_o/c_ in 0.50%, 1.00% and 2.00% PEG-PLO were 92.9%, 89.3% and 94.5%, respectively.

## 3. Discussion

In this study, we synthesized PEGylated-PLO to improve intranasal retentivity and evaluated the physical properties and in vitro permeation and in vivo transnasal absorption enhancement effects.

PLO was modified with PEG using an amine-coupling reaction through NHS [11,16]. PEGylation has been found to be a useful method for prolonging the circulating half-life of proteins in the body [17]. Furthermore, it has been reported that hydrogel having a PEG chain on its surface shows mucoadhesive ability by interaction with the mucosa [18]. As mentioned above, PEG-PLO, which we previously synthesized [11], was difficult to intranasally administer due to its high viscosity. Therefore, we synthesized PEG-PLO with a different MW ratio of PEG and PLO in this study. PEGylation and purification of the product were carried out according to the method previously reported [11]. We confirmed that PLO reacted completely when 10-fold molar PEG was used in a preliminary study. Hence, we could use an ion-exchange spin column to purify the PEG-PLO product (Figure 2).

The PEGylation ratio of PEG-PLO was determined by two methods. The degrees of polymerization of PLO and mPEG-NHS were calculated from a general formula and degree of polymerization. The average PEGylation ratio of PEG-PLO per molecule was calculated by the change in these parameters after PEGylation. The PEGylation ratio of PEG-PLO was 8–9 in both methods, and the NH_2_ content of PEG-PLO was reduced compared with that before PEGylation (Table 2). This suggested that PEG-PLO might decrease the permeation-enhancing titer compared with that of PLO.

The retention time of PEG-PLO on an SSP was significantly prolonged compared with that of PLO (Figure 4), which was consistent with our previous report [11]. The reason for this prolongation was presumed to be caused by an increase in the viscosity by PEGylation [12]. This result suggested that PEGylation might improve the adhesive/retentive properties of HPBAAs and that PEG-PLO possesses high mucoadhesive/retentive properties in vivo as well.

As shown in Figure 5, PEG-PLO has the ability to enhance permeation of hydrophilic macromolecules across Caco-2 cells; however, the enhancing effect of PEG-PLO was lower than that of PLO. This decrease in permeation-enhancing titer had not been observed in a physical mixture of PEG and PLO in a previous report [11]. The decline of the titer of PEG-PLO seemed to be caused by complex factors such as a decrease in NH_2_ content, which plays a key role in the permeation-enhancing effect of HPBAAs because of their positive charge [14] and steric hindrance by PEG chains. Since it may be attenuated, as observed in vitro, the in vivo absorption-enhancing titer of PEG-PLO should also be confirmed. 

A high concentration of PLO caused a decrease in cell viability, but, in contrast, PEG-PLO had no effect on the cell viability even at a concentration showing the same *P*_app_ as that in unmodified-PLO. This result was similar to the previous report [11], supporting the idea that PEG modification attenuates cytotoxicity caused by PLO at high concentrations, and that this cytotoxicity might be caused by a different mechanism to permeation enhancement. 

The effect of PEGylation on the permeation-enhancing mechanisms of HPBAAs was evaluated by immunofluorescent staining of TJ proteins. The fluorescence intensities of TJ proteins at the intercellular space decreased after exposure to PLO, suggesting that the mechanism of hydrophilic macromolecule permeation enhancement by PLO was a result of opening of the paracellular pathway due to the changing localization of TJ proteins, like PLA [6,7]. However, the fluorescence intensity of claudin-4 remained high even after exposure to PLO. Hence, the permeation-enhancing ability of PLO might have greater involvement in occludin than claudin molecules. Furthermore, the fluorescence intensities of TJ proteins after exposure to PEG-PLO also decreased as in the PLO exposure, suggesting that PEGylation did not change the permeation-enhancing mechanisms of HPBAAs.

In order to elucidate the impact of PEGylation of HPBAAs on the transnasal absorption-enhancing effect, *i.n.* administration experiments were performed in a closed system. PEG-PLO also enhanced the transnasal absorption of FD-4 in a dose-dependent manner similar to PLO. Interestingly, the attenuation of absorption-enhancing effect titer by PEGylation in vivo was smaller than that observed in vitro. In general, the absorption-enhancing effect via the epithelium by a cationic absorption enhancer is less in vivo than in vitro at the same concentration. This has been presumed to be due to the neutralization of the positive charge of the cationic absorption enhancer by interaction with the negatively charged part of mucin contained in a large amount of mucus on the mucosa [19,20]. In a study on particle delivery to the mucous membrane [21], it has been reported that particles having a high density of PEG chains became difficult to capture by mucus, and the distribution of the particles to the mucous membrane was improved. For these reasons, the greater transnasal absorption-enhancing effect of PEG-PLO than that assumed from in vitro results was deduced to be due to PEG chains protecting the electrostatic interaction between PLO and mucin, which might cause attenuation of the absorption-enhancing effect of cationic absorption enhancers.

We used an “in vivo intranasal retentivity evaluation system” [14] to evaluate the improvement effect of retentivity in the nasal cavity by PEGylation of PLO. In PLO, the absorption-enhancing effect in the open system remarkably decreased as compared with the closed system. This was considered to be due to the drug solution containing PLO administered to the nasal cavity flowing out from the absorption area in the nasal cavity to the pharynx side, since PLO did not have mucoadhesive/retentive properties. On the other hand, with PEG-PLO, the absorption-enhancing effect in the open system was almost the same as that in the closed system, indicating that retentivity of PLO in the nasal cavity was improved by PEGylation. Although the detailed mechanism is not clear, it was thought to be due to the viscosity of PLO being increased by PEGylation [12] and adhesion to the mucosa being improved due to interaction between PEG chains and the mucosa [17]. However, following PEGylation, it was difficult for the mucus to capture PLO, and consequently, the interaction between the cationic region of PLO and the mucosa, which plays a key role in the absorption-enhancing mechanisms of PLO, was sufficiently introduced [21]. In future, a detailed investigation of the improvement effects of PEGylated-HPBAAs on mucoadhesive/retentive properties will be required.

## 4. Materials and Methods

### 4.1. Materials

Poly-l-ornithine hydrobromide (PLO, MW 44.9 kDa) and FD-4 (MW 3.4 kDa) were purchased from Sigma-Aldrich (St. Louis, MO, USA). α-Succinimidyloxy carbonyl-ω-methoxy polyoxyethylene (mPEG-NHS; SUNBRIGHT ME-100TS, MW 9.8 kDa) and α-mercaptoethyl-ω-methoxy polyoxyethylene (mPEG-SH; SUNBRIGHT ME-100SH; MW 9.2 kDa) were obtained from NOF Corporation (Tokyo, Japan). 2-Morpholinoethanesulfonic acid monohydrate (MES) and MTT were purchased from Dojindo Laboratories (Kumamoto, Japan). TNBS was obtained from Wako Pure Chemical Industries, Ltd. (Osaka, Japan). The cell culture reagents and supplies were purchased from Thermo Fisher Scientific, Inc. (Waltham, MA, USA). All other reagents were of analytical reagent grade.

### 4.2. Antibodies

Mouse anti-occludin (1:120), mouse anti-claudin-4 (1:150), rabbit anti-ZO-1 (1:100), alexa fluor 488 goat anti-rabbit IgG (1:400), alexa fluor 633 goat anti-mouse IgG (1:400) and SlowFade diamond anti-fade mountant were purchased from Thermo Fisher Scientific, Inc. Each antibody was used at the dilutions described above for immunofluorescence-microscopy imaging experiments.

### 4.3. Cell Culture

Caco-2 cells were purchased from the American Type Culture collection (Manassas, VA, USA), and were cultured in Dulbecco’s modified Eagle’s medium (DMEM) supplemented with non-essential amino acids (1%), Gluta MAX-1 (1%), antibiotic-antimycotic (1%) and fetal bovine serum (10%) at 37 °C, under a 5% CO_2_ atmosphere. For experimental use, the cells were seeded on a permeable polycarbonate Transwell membrane (pore size: 0.4 μm, growth area: 1.12 cm^2^, Corning Inc., Corning, NY, USA) at 1.0 × 10^5^ cells/cm^2^, and cultured for 21–28 days.

### 4.4. Synthesis Procedure of PEG-PLO

The synthesis of PEG-PLO was performed as reported previously (Figure 1) [11]. Briefly, PLO solution (2.25 mg/mL) was prepared using MES buffer (0.1 M, pH 7.0), and then mPEG-NHS (49.0 mg) was dissolved in the PLO solution (10 mL). mPEG was bound to PLO by stirring the mixture overnight at room temperature. After the stirring, the cationic compound was eluted from the reaction mixture by using an ion exchange spin column (Strong Cation Exchange Spin Column Maxi, Thermo Fisher Scientific Inc.). The reaction solution was centrifuged at 500× *g* for 30 min with the spin column. The spin column was washed with purification buffer (25 mM sodium acetate buffer, pH 5.5, 10 mL) by centrifugation at 500× *g* for 15 min. Elution buffer (purification buffer +1.0 M NaCl, 5 mL) was added, and centrifugation was carried out at 500× *g* for 5 min. This purification was carried out twice. The eluate was dialyzed (Spectra/Por 7 dialysis membrane, RC, MWCO: 8 kDa, Spectrum Laboratories Inc., Rancho Dominguez, CA, USA) against distilled water (1000 mL) for 24 h, outer water was exchanged 4 times. PEG-PLO was obtained in powder form by freeze-drying the dialysate.

HPLC with a size-exclusion column (PROTEIN KW-803, Shoko Co., Ltd., Tokyo, Japan) and refractive index detector (RI-101, Shoko Co., Ltd.) was used to confirm the synthesis of PEG-PLO. A 0.5 M sodium acetate buffer (pH 4.7) was used as the eluent.

### 4.5. Determination of PEGylation Ratio

Elemental analysis and TNBS assay were performed to determine the PEGylation ratio of PEG-PLO according to reported methods with slight modifications [22,23].

In the elemental analysis, PLO and mPEG-SH aqueous solutions were mixed at each ratio and freeze-dried. These samples were analyzed using a micro-element analyzer (MT-6 CHN Corder, Yanaco Technical Science Co. Ltd., Tokyo, Japan). The C/N ratios of known samples were calculated and used as standards. The C/N ratio in PEG-PLO was calculated in the same manner, and the PEGylation ratio of PEG-PLO was determined from the C/N ratio of the standard.

In the TNBS assay, a sodium tetraborate solution (0.1 M, 140 μL) and TNBS solution (0.1 M, 5 μL) were put into wells of a 96-well plate (Non-treated, flat bottom). The various concentrations of PEG-PLO or PLO (50–200 μg/mL, 60 μL) were added to each well and incubated at 37 °C for 60 min. After incubation, absorbance measurement at 450 nm was performed using a microplate reader (Multiskan Ascent, MTX Lab Systems, Bradenton, FL, USA). The PEGylation ratio of PEG-PLO was determined by calculating the number of amino groups on the side chains from the decrease in absorbance rate of PEG-PLO and unmodified-PLO at the same concentration.

### 4.6. In Vitro Inclined Plate Test

In vitro retentivity of PEG-PLO was evaluated by an inclined plate test, as previously reported, with modifications [24]. A SSP was washed with methanol, dried completely, and inclined at 45°. PEG-PLO and PLO were each dissolved in distilled water to 0.1 *w*/*v*%. Thirty-five microliters of the sample solutions were dropped on the inclined SSP, and the time taken to flow down 3 cm was measured as retention time.

### 4.7. Determination of G_t_

Transepithelial electrical resistance (TEER) was measured using a voltohmmeter (Millicell ERS-2, Merck Corp., Darmstadt, Germany) and the measurement values were converted to ohm·cm^2^ by multiplying the resistance by the surface area of the Transwell membrane. For the experiments, Caco-2 cells were washed and replaced with Hank’s balanced salt solution (HBSS). The various concentrations of PLO and PEG-PLO solutions were added to the apical side of the cells. The TEER values were measured after the application of PLO and PEG-PLO. TEER was converted to membrane conductance (*G*_t_ = 1/TEER, mS/cm^2^), and the ratio of *G*_t_ at 120 min to 0 min (*G*_t_ ratio) was calculated.

### 4.8. FD-4 Permeation Study

The FD-4 permeation study was carried out as reported previously [18]. Caco-2 cells were washed and replaced with HBSS. The solutions containing FD-4 (1.0 mg/mL) and various concentrations of PLO and PEG-PLO were added to the apical side of the cells. Permeation samples were taken from the basal side at predetermined times during the permeation studies. The samples were diluted 60 times with 0.2 M potassium dihydrogen phosphate-sodium borate buffer (pH 8.5). The fluorescence intensity of FD-4 in each sample was measured using a spectrofluorometer (RF-5000PC, Shimadzu, Kyoto, Japan) at excitation and emission wavelengths of 495 and 515 nm, respectively. The apparent permeability coefficient (*P*_app_) of FD-4 was determined by *P*_app_ (cm/s) = *dQ*/*dt*/(*A* × *C*_0_), where *dQ*/*dt* is the steady-state permeation rate (μg/s), *A* is the surface area of the Transwell membrane (cm^2^), and *C*_0_ is the initial concentration of FD-4 in the apical side (μg/mL).

### 4.9. MTT Assay

Cell viability after exposure of PLO and PEG-PLO was determined by MTT assay as reported previously [7,25]. Briefly, various concentrations of PLO and PEG-PLO were added to the apical side of cells. After 120 min, each solution was replaced with MTT reagent (0.5 mg/mL dissolved in DMEM) and incubated at 37 °C for 180 min. The formazan generated from MTT was collected using dimethyl sulfoxide (DMSO), and the absorbance at 540 nm was measured. The cell viability was expressed as a percentage of control cells treated with vehicle (HBSS) only by the same procedure.

### 4.10. Immunofluorescence Microscopic Imaging of TJ Proteins

Immunofluorescence microscopic imaging of TJ proteins was performed as reported previously [26]. Briefly, Caco-2 cells after exposure to PEG-PLO (10 μM) and PLO (1.0 μM) were washed twice with phosphate-buffered saline (PBS) and fixed in acetone-methanol (1:1) at 4 °C for 10 min. After washing twice with PBS, the cells were blocked in PBS containing 0.1% Tween 20 (PBS-T) with 3% bovine serum albumin (BSA) for 60 min at room temperature, and incubated with primary antibodies at 4 °C overnight. After incubation, the cells were washed three times with PBS-T for 10 min, followed by incubation with secondary antibodies for 90 min. The fluorescence images were visualized using a FV1000 confocal laser scanning microscope (CLSM, Olympus, Tokyo, Japan) and the images were analyzed using ImageJ software (NIH) as previously described [7,26]. The fluorescence intensity of TJ proteins was calculated using the Measure function of ImageJ and was expressed as a percentage of the control cells treated with vehicle (HBSS) only by the same procedure. The measurement was performed once for each image.

### 4.11. Animals

Male Wistar rats (8 weeks of age, body weight: 220–270 g) were supplied by Sankyo Labo Service Co., Ltd. (Tokyo, Japan). The animals were provided with food and water ad libitum and kept under a 12/12 h light/dark cycle with at least 7 days of local vivarium acclimatization before experimental use. The rats were fasted for about 16 h before experimental use. All animal experiments were approved by the Institutional Animal Care and Use Committee of Josai University.

### 4.12. Intravenous (i.v.) Injection Study

FD-4 solution (3.3 mg/mL in saline, 3.3 mg/kg) was injected into the left jugular vein of anesthetized rats (urethane 25 *w*/*v*% in saline, 1.2 g/kg, intraperitoneal administration). This study was carried out using the same surgical procedure as that used in the intranasal administration study (described below).

### 4.13. Intranasal (i.n.) Administration Study

The *i.n.* administration study was carried out using an “in vivo intranasal retentivity evaluation system”, i.e., closed and open systems [14]. The rats anesthetized as described above were treated surgically using a previously reported method [27]. A cannula was inserted into the trachea to maintain respiration. In the closed system, an occluded silicone tube was inserted in the esophagus direction of the pharynx to prevent the dosage formulation from leaking to the esophagus side (Figure 8A). In contrast, in order to allow the administered solution to leak from the nasal cavity to the pharynx side in the open system, an unoccluded silicone tube was inserted in the esophagus (Figure 8B). The nasopalatine duct was sealed with medical adhesive to prevent any test solution from leaking into the buccal cavity. The FD-4 solution for *i.n.* administration (33 mg/kg in 0.4 mL/kg) was administered at a distance of 8 mm from the entrance of the left nostril using a microsyringe having a polyethylene tube (diameter: inner 0.5 mm, outer 1.0 mm) attached to the tip. In the open system, the neck and body of the rats were inclined by 30 degrees (total 60 degrees) 10 s after administration in order to allow leakage of dosage formulation from the nasal cavity to the pharynx. Blood samples (0.15 mL) were collected from the right jugular vein using a heparinized syringe and needle at predetermined times after administration, and plasma samples were obtained by centrifugation (15,000 rpm, 4 °C, 5 min, rotation radius: 71 mm). The fluorescence intensity of plasma FD-4 in plasma was measured as mentioned in Section 4.8.

### 4.14. Pharmacokinetic Analysis

The plasma data of FD-4 after *i.v.* administration were analyzed by a non-linear least squares regression program (algorithm: damping Gauss–Newton method). The maximum plasma FD-4 concentration (*C*_max_) and the time to reach the *C*_max_ (*T*_max_) were obtained from the transition of plasma FD-4 concentration. The area under the plasma concentration-time curve from 0–540 min (*AUC*_0–540_) and the area under the first moment curve (*AUMC*_0–540_) were calculated using trapezoidal methods. The mean residence time (MRT) was calculated by moment analysis (*AUMC*_0–540_/*AUC*_0–540_). The maximum absorption rate (MAR) was determined by a deconvolution method. The bioavailability of FD-4 following *i.n.* administration based on the period 0 to 540 min (*F*_0–540_) was calculated. The enhancing ratio (ER) was expressed as the ratio of the *AUC*_0–540_ of each group to the control group. The retentivity improvement rate (*F*_o/c_) was calculated as the ratio of *AUC*_0–540_ in the open system to that in the closed system.

### 4.15. Statistical Analysis

Each datum is represented as the mean or mean ± standard error (S.E.). The two groups were compared using Student’s *t*-test, and multiple comparison was performed using Dunnett’s test. A *p* value of less than 0.05 was regarded as significant.

## 5. Conclusions

We synthesized PEG-PLO with a new MW ratio and evaluated the improvement effect of retentivity in the nasal cavity by PEGylation. PEG-PLO exhibited sufficient transnasal absorption-enhancing ability even in an open system, demonstrating that PEGylation improved the intranasal retentivity of HPBAAs. These findings revealed that a functional absorption enhancer that can sufficiently improve the nasal absorbability of poorly absorbable drugs by increasing the retentivity in the nasal cavity can be synthesized by introducing PEG side chains as a functional component to HPBAAs.

## Figures and Tables

**Table 1:** Measurement values and carbon-to-nitrogen (C/N) ratio of PEG-PLO by elemental analysis.

		**Measurements (%)**			**C/N (Measurement)**	**C/N (Theoretical)**	**Measurement/Theoretical**	**Measurement Deviation**
		**C**	**H**	**N**				
PLO + mPEG-SH								
	1: 5 mixture	39.79	7.20	6.74	5.90	6.02	0.98	0.99
	1: 8 mixture	43.58	7.54	5.23	8.33	8.35	1.00	
	1: 11 mixture	45.05	7.87	4.24	10.63	10.68	1.00	
		**Measurements (%)**			**C/N from Measurement**	**Correcting Value**	**PEG/PLO Ratio**	
		**C**	**H**	**N**				
PEG-PLO		48.35	8.05	5.16	9.37	9.45	8.90	

**Table 2:** Physical properties of PEG-PLO obtained, and elemental analysis calculated through the TNBS assay.

	C/N ratio (Corrected) (Elemental Analysis)	PEGylation Ratio	Containing PEG per Molecule	Molecular Weight (Approximately)	NH_2_ Group per 1 kDa (NH_2_ Contents)	NH_2_ Contents Before the Pegylation	NH_2_ Contents Ratio (After/Before PEGylation)
	Absorbance Ratio (TNBS Assay)						
PEG-PLO	9.45	8.90	8–9	115,000	1.94	5.12	0.38
	0.374	8.96					

**Table 3:** Pharmacokinetic parameters of FD-4 after *i.n.* administration in rats under the closed system.

Route	Dose (mg/kg)	Enhancer		C_max_ (μg/mL)	T_max_ (min)	AUC_0–540_ (μg/mL·min)	MRT (min)	F_0–540_ (%)	ER	MAR (μg/min)
*i.v.*	3.3	-		-	-	1462.9 ± 222.6	-	-	-	-
*i.n.*	33	None (Control)		2.3 ± 0.5	187.5 ± 52.5	902.4 ± 186.2	259.1 ± 16.8	6.2	1.0	2.7
		PLO								
			0.05%	16.5 ± 3.3 *	40.0 ± 10.0 *	3483.2 ± 1283.4	189.5 ± 14.7 *	23.8	3.9	28.4
			0.25%	35.9 ± 3.5 *	60.0 ± 9.5 *	6667.6 ± 1361.1 *	153.8 ± 14.8 *	45.6	7.4	67.3
			0.50%	44.0 ± 4.3 *	82.5 ± 14.4	10470.4 ± 1522.8 *	186.8 ± 14.1 *	71.6	11.6	56.0
		PEG-PLO								
			0.50%	24.4 ± 2.1 *	60.0 ± 0.0 *	5906.6 ± 493.8 *	201.4 ± 4.7 *	40.4	6.5	32.9
			1.00%	29.9 ± 2.3 *	67.5 ± 7.5 *	6064.3 ± 332.7 *	171.7 ± 3.5 *	41.5	6.7	38.4
			2.00%	38.6 ± 5.8 *	70.0 ± 10.1	8813.2 ± 2463.9 *	173.9 ± 18.4 *	60.2	9.8	52.7

Each data represents the mean or mean ± S.E. (*n* = 3–7), * *p* < 0.05 compared with the control group.

**Table 4:** Pharmacokinetic parameters of FD-4 after *i.n.* administration in rats under closed and the open systems.

Enhancer			C_max_ (μg/mL)	T_max_ (min)	AUC_0–540_ (μg/mL·min)	MRT (min)	F_0–540_ (%)	ER	MAR (μg/min)	F_o/c_ (%)
Closed system										
	None (Control)		2.3 ± 0.5	187.5 ± 52.5	902.4 ± 186.2	259.1 ± 16.8	6.2	1.0	2.7	
	PLO	0.25%	35.9 ± 3.5	60.0 ± 9.5	6667.6 ± 1361.1	153.8 ± 14.8	45.6	7.4	67.3	
		0.50%	44.0 ± 4.3	82.5 ± 14.4	10470.4 ± 1522.8	186.8 ± 14.1	71.6	11.6	56.0	
	PEG-PLO	0.50%	24.4 ± 2.1	60.0 ± 0.0	5906.6 ± 493.2	201.4 ± 4.7	40.4	6.5	32.9	
		1.00%	29.9 ± 2.3	67.5 ± 7.5	6064.3 ± 332.7	171.7 ± 3.5	41.5	6.7	38.4	
		2.00%	38.6 ± 5.8	70.0 ± 10.0	8813.2 ± 2463.9	173.9 ± 18.4	60.2	9.8	52.7	
Open system										
	None (Control)		1.8 ± 0.5	80.0 ± 36.7	710.6 ± 135.6	269.9 ± 12.9	4.9	1.0	3.6	78.7
	PLO	0.25%	22.7 ± 0.9 *	60.0 ± 0.0	4448.3 ± 336.5	159.8 ± 7.0	30.4	6.3	37.7	66.7
		0.50%	26.5 ± 4.3 *	60.0 ± 0.0	6020.9 ± 1009.5	189.9 ± 8.8	41.2	8.5	34.1	57.5
	PEG-PLO	0.50%	22.6 ± 3.8	80.0 ± 20.0	5486.5 ± 1904.8	190.7 ± 23.1	37.5	7.7	25.0	92.9
		1.00%	25.4 ± 3.0	60.0 ± 0.0	5416.5 ± 348.6	186.9 ± 11.0	37.0	7.6	36.1	89.3
		2.00%	35.3 ± 2.4	80.0 ± 20.0	8328.3 ± 935.5	182.0 ± 20.9	56.9	11.7	50.7	94.5

Each data represents the mean ± S.E. (*n* = 3–7), * *p* < 0.05 compared with the same concentration in the closed system.
